# Risk Factors for Acute Kidney Injury in Patients Undergoing Total Joint Arthroplasty

**DOI:** 10.3390/reports7040088

**Published:** 2024-10-31

**Authors:** Hazal Nur Kılıc, K. Sanem Cakar Turhan, Suheyla Karadag Erkoc, Merve Hayriye Kocaoglu

**Affiliations:** 1 Department of Anesthesiology and Reanimation, Duzıcı State Hospital, Turkish Ministry of Health, 80600 Osmaniye, Türkiye; hazalnurtezel@yahoo.com; 2 Department of Anesthesiology and Reanimation, Ankara University School of Medicine, 06100 Ankara, Türkiye; sanemcakar@yahoo.com; 3 Department of Anesthesiology and Reanimation, Ankara University School of Dentisty, 06560 Ankara, Türkiye; mervearal@yahoo.com

**Keywords:** postoperative acute kidney injury, total joint arthroplasty, comorbidity, anemia, hypertension

## Abstract

**Objective**: The present study investigates the incidence of postoperative acute kidney injury (AKI) and related risk factors in patients undergoing total joint arthroplasty. **Methods**: Included in the study were patients undergoing joint arthroplasty in 2015–2020. The patients with acute or chronic renal failure were excluded. The participants’ demographical data, American Society of Anesthesiologist (ASA) score, Charlson Comorbidity Index (CCI), type of operation, duration of surgery, presence of comorbidities, preoperative anemia, preoperative albumin levels, use of nephrotoxic agents, number of transfusions during perioperative period, presence of AKI according to Kidney Disease Improving Global Outcome (KDIGO) scores, and length of hospital and intensive care unit stay were evaluated. **Results:** The study was initiated with 1780 patients: 113 patients were excluded due to chronic kidney failure, 108 patients were excluded due to acute kidney failure, 648 patients were excluded because their data could not be reached, and finally, 911 patients were included in the study. AKI was detected in 134 patients (14.7%), and the number of patients in the KDIGO1 and KDIGO2 groups were 120 and 14, respectively. When evaluated according to the variable significance test result and clinical significance, the model consists of variables such as ASA, CCI, hypertension, nonsteroidal anti-inflammatory drugs (NSAIDs), vancomycin, beta lactam, contrast material and preoperative anemia, operation type, and anesthesia management. Machine learning analyses were performed using 11 variables (10 independent and 1 dependent variable). Logistic regression, naive Bayes, multilayer perceptron, bagging, and random forrest approaches were used for evaluation of the predictive performance. In an evaluation of the true classification ratio, the best result was obtained with the logistic regression method at 85.2%. **Conclusions:** The study revealed advanced age, high ASA and CCI, presence of diabetes and hypertension, NSAID, vancomycin and contrast material, and the presence of preoperative anemia to be independent risk factors for AKI.

## 1. Introduction

Hip and knee osteoarthritis are among the most common reasons for joint pain, leading to severe restrictions in daily or sportive activities in adults. Although the primary approach to such conditions is conservative treatment, arthroplasty surgery is available as a final option in those with resistant pain and functional loss and with radiologically diagnosed end-stage osteoarthritis.

Recent advances in postoperative surgical care have led to faster recovery, early mobilization, and reduced hospital stays in patients following total joint arthroplasty, although complications such as acute kidney injury can be detrimental to the healing process. The global incidence of acute kidney injury (AKI) is 22%, which increases to 57% in intensive care units. The lack of consensus on the prediction and determination of the risk factors for the development of AKI in this group of patients complicates effective prognosis and preoperative optimization in arthroplasty surgery [1]. Patients who develop AKI in the postoperative period are at greater risk of morbidity and mortality, and while there are several perioperative factors associated with AKI in patients undergoing arthroplasty, some risk factors remain uncertain [2]. The incidence of AKI following arthroplasty surgery reported in previous studies covers a wide range, from 1.3% to 21%, and the prognosis is poor, progressing to chronic renal disease (CRD), end-stage renal failure, and cardiovascular disease in the later period and, finally, death [3,4,5,6,7]. Given the lack of an effective treatment for AKI, the early determination of the risk factors, preventive measures, and early interventions remain the optimum approach. Traditional risk factors for AKI are advanced age; comorbidities such as previous CRD; sepsis; and exposure to nephrotoxic agents (nonsteroidal anti-inflammatory drugs, vancomycin, aminoglycosides, cisplatin, methotrexate, amphotericin B, colistin, tenofovir, and contrast material). Recently, new risk factors for AKI have been defined, including hyperuricemia, hypoalbuminemia, obesity, anemia, and hyperglycemia. The basic mechanisms linking these unconventional risk factors with AKI, and whether or not the optimization of these risk factors decreases the development of AKI, are uncertain [3,4,5,6,7].

The present study investigated the incidence of postoperative acute kidney injury and the related risk factors in patients undergoing total joint arthroplasty.

## 2. Materials and Methods

The present study was conducted retrospectively at a single center and was approved by the Institutional Ethics Committee (ethical committee approval number: I05-310-22, date: 30 May 2022), and written informed consent was obtained from all participants. Included in the study were patients who underwent joint arthroplasty between 2015 and 2020 in the Department of Orthopedics and Traumatology of the Faculty of Medicine of XX University. Given the retrospective and descriptive design of the study, the required sample size was not calculated. At the outset, the files of patients aged ≥18 years who had undergone total joint arthroplasty between 2015 and 2020 were screened and subjected to data analysis. Excluded from the study were patients with acute or chronic renal failure, those below the age of 18 years, and those whose data could not be accessed. The files of the remaining participants were evaluated for demographical data, comorbidity scores, type of surgical procedure, duration of surgery, anesthesia method, comorbidities, perioperative drug and contrast material use, preoperative laboratory values, presence of intraoperative hypotension, perioperative transfusion amounts, duration of hospitalization and intensive care unit stay, creatinine, GFR, and Kidney Disease Improving Global Outcome (KDIGO) scores at the postoperative 24th hour and 48th hour and on the 7th day (or discharge values for those hospitalized for fewer than 7 days).

AKI is defined by KDIGO as an increase in serum creatinine ≥0.3 mg/dl (≥26.5 µmol/L) within 48 h or an increase in the assumed creatinine value of ≥1.5-fold or urine output <0.5 mL/kg/h for 6 h [8].

AKI was staged as follows: Stage I: increase in serum creatinine of 1.5 to 1.9 times baseline, an increase in serum creatinine by ≥0.3 mg/dL (≥26.5 micromole/L), or a reduction in urine output to <0.5 mL/kg/h for 6–12 h; Stage II: increase in serum creatinine of 2.0 to 2.9 times baseline or a reduction in urine output to <0.5 mL/kg/h for ≥12 h; Stage III: increase in serum creatinine of 3.0 times baseline, an increase in serum creatinine to ≥4.0 mg/dL (≥353.6 micromole/L), a reduction in urine output to <0.3 mL/kg/h for ≥24 h or anuria for ≥12 h, the initiation of kidney replacement therapy, or, in patients <18 years, a decrease in the estimated glomerular filtration rate (eGFR) to <35 mL/min/1.73 m^2^ [9].

The CKD-EPI formula was used to calculate the GFR. A total of 1780 patients were screened with a diagnosis of joint arthroplasty, of whom 113 were excluded due to chronic kidney failure and 108 due to acute kidney failure, while 648 were excluded due to inaccessible data. Finally, 911 patients were included in the study.

SPSS (Version 11.5, Chicago, IL, USA, SPSS Inc.) was used for data analysis. Quantitative variables were presented as descriptive statistics, mean ± standard deviation, and median (min–max) values; the qualitative variables were patient numbers (percentages). A Mann–Whitney U test was used for variables without a normal distribution assumption for the evaluation of any difference between categories of qualitative variables that has 2 categories in terms of quantitative variables. Chi-square and Fisher exact tests were used for the analysis of correlations between two qualitative variables, and a univariate logistic regression test was used to determine the risk factors for AKI. Statistical significance was set as *p* < 0.05.

The importance of variables was evaluated with gain ratio and information gain tests. Logistic regression, naive Bayes, multilayer sensor, bagging, and random forest were used as the machine learning classification methods. The data set was tested using 10× cross-validation, and the results were obtained after repeating all analyses 1000 times. The Correct Classification Rate (CCR), F-criteria, ROC area, and precision–recall area were used for the performance criteria. All analyses were performed using the R programming language, benefiting from the RWeka and e1071 packets within the R programming language.

## 3. Results

There were 911 patients included in the study. Of the patients, 667 (73.2%) were female and 244 (26.8%) were male, and the mean age was 64.68 ± 12.36. AKI was observed in 134 patients, with an incidence of 14.7%. When the patients with AKI and without AKI were compared in terms of demographical variables, no significant difference was noted in terms of gender or BMI, whereas a significant difference was noted in terms of age, Charlson Comorbidity Index (CCI), American Society of Anesthesiologists (ASA) score, and operation type. Age and CCI were higher in patients who developed AKI. AKI developed in 5.2% of the ASA I patients, 13.2% of the ASA II patients, and 22.7% of the ASA III patients. The highest AKI ratio was in the patients who underwent total revision hip arthroplasty (27%), while the lowest AKI ratio was observed in those who underwent partial revision hip arthroplasty (9.5%) (Table 1).

Diabetes mellitus (DM) was noted in 25.5% of the samples, hypertension (HT) in 52.8%, coronary artery disease (CAD) in 17.8%, and congestive heart failure (CHF) in 7%. A statistically significant difference was noted between the groups in terms of the presence of diabetes mellitus, hypertension, vancomycin and contrast material use, and preoperative anemia. The ratios of AKI in patients with DM was 20.7% and 12.7% in patients without DM, and the ratio of AKI in patients with HT was 20% and 8.8% in those without HT. The incidence of AKI in patients using vancomycin was 30.3% and 13.5% in patients not using vancomycin. The ratio of AKI in patients using contrast material was 25.6% and 12.2% in patients not using contrast material. Finally, the ratio of AKI in patients with and without preoperative anemia was 17.8% and 12%, respectively (Table 2).

There was a statistically significant difference between the groups in terms of preoperative albumin levels, basal GFR, erythrocyte suspension transfusion amounts, and durations of hospitalization. The preoperative albumin level in the patients with AKI (37.74 ± 5.23) was significantly lower than in those without AKI (39.16 ± 5.39), the basal GFR value was significantly lower in the patients with AKI, and the amount of erythrocyte suspension transfusion and the mean duration of hospitalization were higher in the patients with AKI. ICU admission was found at 5% in all patients, and the presence of AKI had no significant effect on this value.

The number of patients with AKI at the postoperative 24th hour in KDIGO groups 1 and 2 were 42 and 4, respectively. At the postoperative 48th hour, the number of patients in the KDIGO 1 and KDIGO 2 groups increased to 99 and 13, respectively. During hospitalization, AKI was detected in 134 patients (14.7%). The number of patients in the KDIGO 1 and KDIGO 2 groups were 120 (89.6%) and 14 (10.6%), respectively.

Seventy-one patients were discharged with a diagnosis of AKI. Sixty-five (90.3%) were in KDIGO group 1, and six (9.7%) were in KDIGO group 2. Furthermore, two patients who were in KDIGO group 1 at the postoperative 48th hour progressed to KDIGO group 2 and were discharged as KDIGO 2. None of the patients required renal replacement therapy.

Univariate logistic regression analysis revealed that age, ASA, CCI, diabetes mellitus, hypertension, vancomycin, contrast material, and preoperative anemia were significant risk factors for AKI. A 1 unit increase in age resulted in a 1.04-fold increase in AKI risk; a 2.917-fold increase in AKI risk was noted between ASA physical statuses I and II, while a 5.352-fold increase in AKI risk was noted between ASA physical status III and I. The presence of diabetes mellitus, hypertension, vancomycin use, contrast material use, and preoperative anemia resulted in 1.799-fold, 2.572-fold, 2.788-fold, 2.479-fold, and 1.583-fold increases in AKI risk, respectively (Table 3).

**Figure 1:**
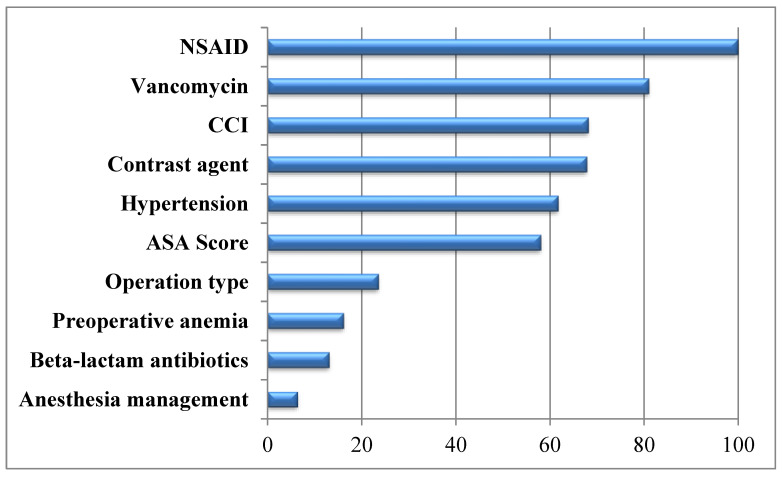
Variable significance results for the gain ratio method.

The significance of variables and the effect of these variables on AKI development were evaluated by using gain ratio variable significance test in Figure 1. When evaluated according to the variable significance test result and clinical significance, the model consists of variables such as ASA, CCI, hypertension, nonsteroidal anti-inflammatory drugs (NSAIDs), vancomycin, beta lactam, contrast material, preoperative anemia, operation type, and anesthesia management. As a result, 11 variables (10 independent and 1 dependent variable) were included to the study, and machine learning analysis was performed by using these variables.

**Figure 2:**
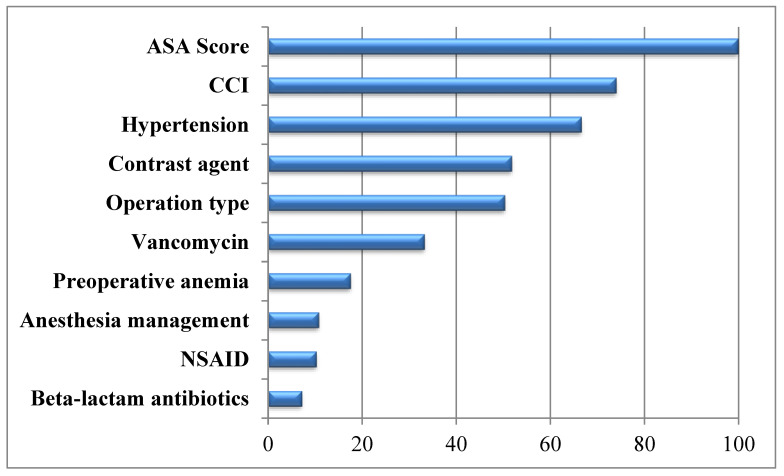
Variable significance results for the information gain method.

The significance of variables and the effect of these variables on AKI development were evaluated by using the information gain variable significance test in Figure 2. When evaluated according to the variable significance test result and clinical significance, the model consists of variables such as ASA, CCI, hypertension, nonsteroidal anti-inflammatory drugs (NSAIDs), vancomycin, beta lactam, contrast material, preoperative anemia, operation type, and anesthesia management.

**Figure 3:**
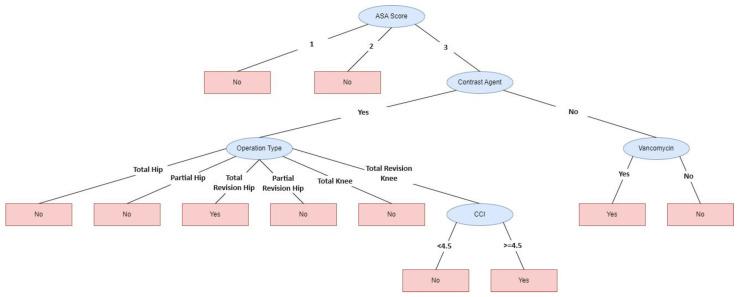
Tree diagram. 1: ASA score 1, 2: ASA Score 2, 3: ASA score 3.

When the two methods were evaluated, 11 variables (10 independent and 1 dependent variable) were included in the study, and machine learning analysis was performed by using these variables (Figure 3). According to Figure 3, AKI risk increased in patients with ASA score III, using contrast material and undergoing total revision knee arthroplasty and total revision hip arthroplasty with a Charlson Comorbidity Index score ≥ 4.5. AKI risk increased in patients with an ASA score III not using contrast material but using vancomycin.

Logistic regression, naive Bayes, multilayer perceptron, bagging, and random forrest approaches were used for the evaluation of predictive performance. In an evaluation of the true classification ratio, the best result was obtained with the logistic regression method at 85.2%. In other words, the method was 85.2% effective in identifying those with and without AKI (Table 4).

## 4. Discussion

Arthritis is one of the leading problems encountered by the aging population around the world, with pain and loss of function being identified as significant problems. Although the need for arthroplasty surgery has decreased as a result of the recently developed medications, arthroplasty surgery still maintains importance as a treatment modality. The increased comorbidities and multidrug use associated with aging make older adults more susceptible to perioperative complications, and postoperative management becomes more difficult. The sufficiency of renal perfusion during the perioperative period is especially important in the older adult population with several comorbidities, and a deterioration in renal perfusion during the perioperative period can lead to acute kidney injury and even chronic renal failure if not spotted. The worldwide incidence of AKI is 22% and 57% in intensive care units, making AKI a global health problem. In the present study, we investigated the risk factors associated with postoperative acute kidney injury (AKI), as well as approaches to the prevention of AKI through control of the identified risk factors [1,2,3,4,5,6,7].

The Charlson Comorbidity Index, developed by Mary Charlson et al., in 1987, is a scoring system that determines the 1-year mortality of patients from the time of hospital admission and is important not only for the determination of mortality but also for the rating of comorbidities in terms of complications [10].

In a study by Hancı et al., evaluating the risk factors associated with major orthopedic surgery, a relationship was identified between the Charlson Comorbidity Index and postoperative AKI [11]. Soohoo et al.’s investigation of complication rates following total knee arthroscopy revealed that patients scoring 3 or higher from the Charlson Comorbidity Index were at greater risk of postoperative infection, and the same study reported that an increase of 0 to 1 in the Charlson Comorbidity Index coincided with a 170% increase in mortality rates [12]. In a study by Wong et al., investigating postoperative complications in cases of femur neck fracture, the age-adjusted Charlson Comorbidity Index of patients who developed complications (4.88 ± 1.44) was significantly higher than in those without complications (3.99 ± 1.37) [10]. Similar to previous studies, in the present study, the Charlson Comorbidity Index was found to be significantly high in the patients who developed postoperative AKI (3.60 ± 1.53). Furthermore, the results of a logistic regression analysis revealed a high CCI to be a significant risk factor for AKI.

Advanced age is a significant risk factor for the development of AKI due to the several associated age-related physiological changes and comorbidities that develop. In a study by Nikkinen et al., investigating AKI in patients undergoing lower extremity arthroplasty surgery, the mean age of those who developed AKI was found to be significantly high [13]. In a study by Singh et al., involving patients undergoing primary total hip arthroplasty, it was found that, after adjusting for age, AKI was associated with a higher odds ratio of such in-hospital complications as infection, transfusion, revision, and death; a total hospital charge above the median; hospitalization exceeding 3 days; and discharge to a rehabilitation facility [4]. In Privratsky et al.’s study of the association between age and gender and postoperative AKI, the risk of postoperative AKI was found to be higher in those of both sexes aged 50 years or above, with the highest incidence of AKI recorded in the female patients aged 50 years and above [14]. Ferguson et al., reported a 1.62-fold increased risk of AKI for each additional decade [15]. Age was also identified as a significant risk factor for AKI development in the present study, with each 1-unit increase in age increasing the risk of AKI 1.04-fold.

The ASA physical status score measures the presence and degree of comorbidities in patients, assessing patient fitness on a scale of 1 to 4. In a study by Jämsä et al., of patients undergoing arthroplasty surgery, the ASA physical status score was identified as an independent risk factor for AKI, while, in Hancı et al.’s investigation of the risk factors associated with major orthopedic surgery, a correlation was noted between the ASA physical status score and postoperative AKI [6,11]. Mckeag et al., reported that patients with two or more comorbidities were at a significantly greater risk of AKI [16]. In the present study, a statistically significant difference was noted between the ASA physical status scores of the patients with and without AKI, with AKI identified in 5.2% of the patients with an ASA I physical status score, 13.2% of the patients with an ASA II physical status score, and 22.7% of the patients with an ASA III physical status score. An ASA physical status score was thus identified as an independent risk factor for AKI. In the present study, the risk of AKI in patients with an ASA physical status of II was 2.917 times greater than in patients with an ASA physical status of I, while the risk of AKI in patients with an ASA physical status score of III was 5.352 times greater than in those with an ASA physical status score of I.

Uncontrolled hyperglycemia and diabetes mellitus are well known to cause end-organ damage, with those associated with diabetes mellitus in particular being cardiac diseases, vasculopathy, neuropathy, retinopathy, and nephropathy. Diabetic nephropathy is a frequent complication of diabetes mellitus. Takeshita et al., reported the presence of diabetes mellitus to be a significant risk factor for AKI, while Warth et al., reported that patients who developed AKI were more likely to be obese and to have diabetes mellitus [17,18]. In the study by Choi et al., diabetes mellitus was identified as a risk factor in their multivariant analysis [19]. In the present study, the incidence of AKI was found to be significantly high in patients with diabetes mellitus (20.7%), while a univariate logistic regression analysis found that the presence of diabetes mellitus increased the rate of AKI 1.799-fold. This finding is correlated with the previous studies, and this is a significant strength of our study. Therefore, we suggest that one of the important factors that should be focused on during the perioperative planning of patients undergoing arthroplasty could be tight glucose control.

Hypertension is a leading risk factor for morbidity and mortality around the globe and is associated with such end-organ conditions as vasculopathy, cardiac disease, cerebrovascular injury, and nephropathy. In a study by Jafari et al., of patients undergoing total joint arthroplasty, hypertension was found to be an independent risk factor for the development of AKI [20]. Weingarten et al., on the other hand, reported that the risk of AKI increases in line with the number of antihypertensive medications being used, which may be attributable to the association between the use of multiple antihypertensive medications and uncontrolled hypertension [21]. In the study by Challagundla et al., investigating the relationship between antibiotic prophylaxis and AKI, hypertension alone was found to be correlated with AKI, although no differences were detected in the development of AKI, depending on the variations in antibiotics [22]. In the present study, the number of hypertensive patients was found to be high among those who developed AKI, and a univariate logistic regression analysis found the presence of hypertension to increase the risk of AKI 2.572-fold. There are several modalities related to prophylactic antibiotic use in patients undergoing arthroplasty. The effect of surgical prophylaxis on renal function is an important issue in patients undergoing arthroplasty, as they tend to be of advanced age and have several comorbidities. In the study by Ferguson et al., of 43 patients undergoing primary hip and knee arthroplasty, surgical prophylaxis with a single dose of gentamycin was reported to have no effect on the development of postoperative AKI [15]. In the study by Jämsä et al., in which gentamycin was added to the cement of 94% of the patients, a lower rate of AKI development was reported in these patients [6]. In the present study, a single dose of gentamycin given to patients undergoing arthroplasty did not lead to a significant increase in the rate of postoperative AKI. Another antibiotic that is frequently used in arthroplasty surgery is vancomycin, given either by the intravenous route or mixed with the cement. In a study by Courtney et al., a significant increase in the incidence of postoperative AKI was noted in the patients who underwent primary joint arthroplasty under prophylaxis with cefazoline and vancomycin in combination [23]. A study by Branch-Elliman et al., compared vancomycin and the beta-lactam group, as well as their use in combination, and reported the rate of AKI to be highest in the combination group [24]. In a study by Klasan et al., it was reported that the regional administration of intraosseous vancomycin did not contribute to an increase in the risk of vancomycin-related complications, including AKI [25]. In the present study, the rate of AKI was found to be high in patients treated with vancomycin, which was demonstrated to increase the risk of AKI 2.788-fold. This is a very valuable data. The patients undergoing arthroplasty surgery are at advanced ages and have several comorbidities, so antibiotic prophylaxis should be done carefully, and close monitorization of perioperative renal functions is essential. Several investigations involving the use of oral or intravenous contrast materials were made with patients undergoing arthroplasty during the perioperative period for various reasons. As such patients tend to be of an advanced age, the primary concern should be focused on renal function protection. In a study by Kim et al., the use of contrast materials in patients undergoing gastric surgery was found to significantly increase AKI development [26]. In the present study, the ratio of AKI in patients using contrast material was 25.6% and 12.2% in patients not using contrast material. Thus, sufficient perioperative hydration of these patients is very important for the prevention of contrast material-induced AKI. To our knowledge, in the literature, there is no study investigating contrast-induced AKI in patients undergoing arthroplasty surgery.

Hospitalization is prolonged in patients that develop AKI, leading to an increase in the associated healthcare costs. In the study by Nadkarni et al., the mean duration of hospitalization was reported to be 5 days or more in patients who developed AKI [27]. In Warth et al.’s study of patients undergoing arthroplasty surgery, the mean duration of hospitalization was 1.8 days longer in patients who developed AKI [18]. Similar to previous studies, the mean duration of hospitalization in the present study was significantly higher in the patients who developed AKI, while no significant difference was noted in ICU stays. Many other factors could also prolong hospitalization, such as surgical complications and deterioration of present comorbidities. However, as the current study is a retrospective study, these factors could not be evaluated and included in the analysis. Further prospective studies are needed to evaluate the causes contributing to increased length of stay.

In the study of Abar et al., patients who underwent bilateral joint arthroplasty in the same session were at significantly greater risk of developing AKI than those who underwent unilateral joint arthroplasty [28]. In the study by Yadav et al., no significant difference was noted between revision and primary arthroplasty surgeries in terms of AKI development, although AKI was more common in those undergoing revision arthroplasty, in which a cement containing an antibiotic was used [29]. In the present study, the highest rate of AKI was noted in the patients undergoing total revision hip arthroplasty (*p* = 0.002), which may be due to increased bleeding associated with total revision hip arthroplasty compared to other arthroplastic procedures and the lower bleeding rates associated with knee arthroplasties due to the application of a tourniquet. Thus, the amount of erythrocyte suspension transfusion was found to be high in patients developing AKI, which also supports the highest rate of AKI in patients undergoing total revision hip arthroplasty. As well known, bleeding is expected to be high in revision arthroplasties, but the data related to the amount of bleeding and its correlation with AKI development are not included in the analysis of the current retrospective study. This is another limitation of the current study. In our clinic, tranexamic acid is administered to all patients undergoing arthroplastic procedures as a hemostatic agent, and for knee arthroplasties, tourniquet application is also completed.

In the study by Weingarten et al., postoperative AKI was found to be significantly more common in patients using intraoperative NSAIDs [21]. In the study by Mittal et al., the rate of AKI was found to be 2.9% with use of dual NSAIDs in patients undergoing total joint arthroplasty [30]. NSAIDs are widely used analgesic drugs in our population, and 98.8% of the cohort studied used NSAIDs. Thus, the incidence of AKI might be lower in populations that do not regularly use NSAIDs. In the present study, 98.8% of the patients were using NSAIDs. Although this may lead to difficulties in statistical analyses of the effects of NSAIDs on AKI development, the presence of the data related to NSAID use in all patients developing AKI carried this parameter to a significant point. An evaluation of the parameters found to be predictive of AKI revealed the most significant to be NSAID use in the present study. Thus, close perioperative monitorization of renal function is necessary in patients undergoing total joint arthroplasty if NSAIDs are used for analgesia.

The current study is a retrospective study. Thus, it has some limitations. The number of antihypertensive medications and the data related to whether hypertension is controlled or uncontrolled could not be obtained from the patients’ files. Another limitation is related to NSAID use. As already mentioned, NSAID use was found to be correlated with AKI development, but as the study is retrospective, subdivision of the NSAIDs could not be done. In addition, the time of NSAID administration, whether preoperatively or postoperatively, could not be obtained from the patients’ files. The data related to the amount of bleeding and its correlation with AKI development are not included in the analysis, which is another limitation of the current study. Follow-up prospective studies are necessary to further determine the risk factors for AKI in patients undergoing arthroplasty surgery.

## 5. Conclusions

The study revealed advanced age, high ASA and CCI, the presence of diabetes and hypertension, NSAIDs, vancomycin and contrast material, and the presence of preoperative anemia to be independent risk factors for AKI.

## Figures and Tables

**Table 1:** Demographical variables.

Variables		AKI			
		Absent	Present	Total	*p*-Value
**Gender, *n* (%)**	Female	569 (85.3)	98 (14.7)	667 (73.2)	0.982 ^a^
	Male	208 (85.2)	36 (14.8)	244 (26.8)	
**Age**	Mean ± SD	63.90 ± 12.34	69.19 ± 11.51	64.68 ± 12.36	**<0.001 ^c^**
	Median (Min–Max)	65.00 (21.00–94.00)	70.00 (28.00–98.00)	66.00 (21.00–98.00)	
**BMI**	Mean ± SD	30.09 ± 6.06	31.08 ± 5.73	30.24 ± 6.02	0.078 ^c^
	Median (Min–Max)	29.72 (14.69–52.24)	30.45 (20.03–44.06)	30.00 (14.69–52.24)	
**Cigarette use, *n* (%)**	Present	192 (88.9)	24 (11.1)	216 (23.7)	0.087 ^a^
	Absent	585 (84.2)	110 (15.8)	695 (76.3)	
**CCI**	Mean ± SD	2.79 ± 1.67	3.60 ± 1.53	2.91 ± 1.67	**<0.001 ^c^**
	Median (Min–Max)	3.00 (0.00–13.00)	3.00 (0.00–9.00)	3.00 (0.00–13.00)	
**ASA score, *n* (%)**	1	201 (94.8)	11 (5.2)	212 (23.4)	**<0.001 ^a^**
	2	332 (86.2)	53 (13.8)	385 (42.5)	
	3	239 (77.3)	70 (22.7)	309 (34.1)	
**Type of operation *n* (%)**	Total Hip Arthroplasty	215 (89.6)	25 (10.4)	240 (26.3)	**0.002 ^a^**
	Partial Hip Arthroplasty	75 (78.1)	21 (21.9)	96 (10.5)	
	Total Revision Hip Arthroplasty	27 (73.0)	10 (27.0)	37 (4.1)	
	Partial Revision Hip Arthroplasty	19 (90.5)	2 (9.5)	21 (2.3)	
	Total Knee Arthroplasty	389 (86.8)	59 (13.2)	448 (49.2)	
	Total Revision Knee Arthroplasty	52 (75.4)	17 (24.6)	69 (7.6)	
**Duration of surgery (min)**	Mean ± SD	235.28 ± 92.62	239.06 ± 88.52	235.84 ± 91.99	0.436 ^c^
	Median (Min–Max)	213.00 (112.00–456.00)	212.50 (123.00–397.00)	213.00 (112.00–456.00)	
**Anesthesia Method, *n* (%)**	General	157 (83.5)	31 (16.5)	188 (20.6)	0.167 ^a^
	Spinal	340 (83.7)	66 (16.3)	406 (44.6)	
	Combined	280 (88.3)	37 (11.7)	317 (34.8)	
**Preoperative Albumin**	Mean ± SD	39.16 ± 5.39	37.74 ± 5.23	38.90 ± 5.39	**0.002 ^c^**
	Median (Min–Max)	40.00 (13.00–57.00)	39.00 (22.00–50.00)	40.00 (13.00–57.00)	
**ES**	Mean ± SD	1.72 ± 1.37	2.00 ± 1.18	1.77 ± 1.34	**0.032 ^c^**
	Median (Min–Max)	1.00 (1.00–11.00)	2.00 (1.00–5.00)	1.00 (1.00–11.00)	
**Duration of hospitalization**	Mean ± SD	7.90 ± 6.92	9.96 ± 8.99	8.20 ± 7.30	**0.001 ^c^**
	Median (Min–Max)	6.00 (3.00–150.00)	7.00 (3.00–74.00)	7.00 (3.00–150.00)	
**Duration of ICU stay**	Mean ± SD	2.79 ± 4.87	3.00 ± 3.77	2.84 ± 4.58	0.611 ^c^
	Median (Min–Max)	1.00 (1.00–22.00)	1.00 (1.00–13.00)	1.00 (1.00–22.00)	

SD: standard deviation, Min: minimum, Max: maximum, ^a^: chi-square test, ^c^: Mann–Whitney U test, BMI: Body Mass Index, CCI: Charlson Comorbidity Index, ASA: American Society of Anesthesiologists, AKI: acute kidney injury, and ICU: intensive care unit.

**Table 2:** Comparison of patients with and without AKI in terms of comorbidities and drug use.

Variables		AKI			
		Absent	Present	Total	*p*-Value
**Diabetes mellitus, *n* (%)**	Present	184 (79.3)	48 (20.7)	232 (25.5)	**0.003 ^a^**
	Absent	593 (76.3)	86 (12.7)	679 (74.5)	
**Hypertension, *n* (%)**	Present	385 (80.0)	96 (20.0)	481 (52.8)	**<0.001 ^a^**
	Absent	392 (91.2)	38 (8.8)	430 (47.2)	
**CAD, *n* (%)**	Present	130 (82.3)	28 (17.7)	158 (17.3)	0.240 ^a^
	Absent	647 (85.9)	106 (14.1)	753 (82.7)	
**CHF, *n* (%)**	Present	54 (84.4)	10 (15.6)	64 (7.0)	0.830 ^a^
	Absent	723 (85.4)	124 (14.6)	847 (93.0)	
**NSAIDs, *n* (%)**	Present	766 (85.1)	134 (14.9)	900 (98.8)	0.383 ^b^
	Absent	11 (100.0)	0 (0.0)	11 (1.2)	
**Aminoglycosides, *n* (%)**	Present	78 (88.6)	10 (11.4)	88 (9.7)	0.351 ^a^
	Absent	699 (84.9)	124 (15.1)	823 (90.3)	
**Beta Lactam, *n* (%)**	Present	79 (79.8)	20 (20.2)	99 (10.9)	0.102 ^a^
	Absent	698 (86.0)	114 (14.0)	812 (89.1)	
**PPI, *n* (%)**	Present	595 (84.6)	108 (15.4)	703 (77.2)	0.306 ^b^
	Absent	182 (87.5)	26 (12.5)	208 (22.8)	
**Vancomycin, *n* (%)**	Present	46 (69.7)	20 (30.3)	66 (7.2)	**<0.001 ^a^**
	Absent	731 (86.5)	114 (13.5)	845 (92.8)	
**Contrast material, *n* (%)**	Present	128 (74.4)	44 (25.6)	172 (18.9)	**<0.001 ^a^**
	Absent	649 (87.8)	90 (12.2)	739 (81.1)	
**Preop Anemia, *n* (%)**	Present	346 (82.2)	75 (17.8)	421 (46.2)	**0.014 ^a^**
	Absent	431 (88.0)	59 (12.0)	490 (53.8)	

^a^: chi-square test, ^b^: Fisher exact test, AKI: acute kidney injury, CAD: coronary artery disease, CHF: congestive heart failure, NSAIDs: nonsteroidal anti-inflammatory drugs, and PPI: proton pump inhibitor.

**Table 3:** Results of univariate binary logistic regression analysis for AKI risk factors.

Variables (Reference)		β	SD	*p* Value	Odds Ratio	95% CI for Odds Ratio	
						Min	Max
**Gender (Female)**	Male	0.005	0.211	0.982	1.005	0.664	1.520
**Age**		0.039	0.009	**<0.001**	1.040	1.022	1.058
**BMI (≤35)**	>30	0.309	0.214	0.148	1.363	0.896	2.071
**ASA (1)**	2	1.071	0.343	**0.002**	2.917	1.489	5.715
	3	1.677	0.338	**<0.001**	5.352	2.758	10.384
**CCI**		0.272	0.054	**<0.001**	1.313	1.181	1.459
**Duration of surgery**		0.001	0.001	0.660	1.001	0.998	1.002
**Intraoperative Hypotension (Absent)**	Present	0.213	0.200	0.287	1.238	0.836	1.833
**Anesthesia Method** **(Combined)**	General	0.402	0.263	0.127	1.494	0.892	2.503
	Spinal	0.385	0.221	0.081	1.469	0.953	2.264
**Type of operation (Total Knee Arthroplasty)**	Other	0.243	0.188	0.198	1.274	0.881	1.843
**Diabetes mellitus (Absent)**	Present	0.587	0.199	**0.003**	1.799	1.218	2.657
**Hypertension (Absent)**	Present	0.945	0.205	**<0.001**	2.572	1.722	3.842
**CAD (Absent)**	Present	0.274	0.233	0.241	1.315	0.832	2.076
**CHF (Absent)**	Present	0.077	0.358	0.830	1.080	0.536	2.177
**NSAIDs (Absent)**	Present	-	-	-	-	-	-
**Aminoglycosides (Present)**	Yok	0.325	0.350	0.353	1.384	0.697	2.746
**Beta-Lactam (Absent)**	Present	0.438	0.270	0.104	1.550	0.913	2.631
**PPI (Absent)**	Present	0.239	0.234	0.307	1.271	0.803	2.011
**Vancomycin (Absent)**	Present	1.025	0.286	**<0.001**	2.788	1.591	4.885
**Contrast material (Absent)**	Present	0.908	0.208	**<0.001**	2.479	1.649	3.725
**Preop Anemia (Absent)**	Present	0.460	0.188	**0.015**	1.583	1.05	2.291
**FFP (Absent)**	Present	0.151	0.639	0.813	1.163	0.332	4.074
**ES (Absent)**	Present	0.309	0.205	0.132	1.362	0.911	2.036

β: Beta, SD: standard deviation, CI: confidence interval. AKI: acute kidney injury, CAD: coronary artery disease, CHF: congestive heart failure, NSAIDs: nonsteroidal anti-inflammatory drugs, PPI: proton pump inhibitors, FFP: fresh frozen plasma, and ES: erythrocyte suspension.

**Table 4:** Machine learning results for chronic renal failure.

Method	Performance Criteria			
	CCR	F-Criteria	PRC Area	ROC Area
**Logistic Regression**	0.852	0.794	0.821	0.671
**Naïve Bayes**	0.832	0.817	0.829	0.688
**Multilayer Perceptron**	0.824	0.801	0.787	0.606
**Bagging**	0.850	0.791	0.809	0.649
**Random Forest**	0.824	0.790	0.797	0.616

CCR: Correct Classification Rate, and PRC: Precision–recall curve.

## Data Availability

The data that support the findings of this study are available upon reasonable request from the corresponding author, S.K.E. The data are not publicly available due to containing information that could compromise the privacy of the study participants.
